# Frequency of Founder Mutations in *BRCA1* and *BRCA2* Genes in Hereditary Breast Cancers in Poland vs. Other Countries

**DOI:** 10.3390/cancers18030492

**Published:** 2026-02-02

**Authors:** Beata Kulikowska, Barbara Panasiuk, Renata Posmyk

**Affiliations:** 1 Department of Clinical Genetics, Medical University of Bialystok, 15-089 Bialystok, Poland; barbara.panasiuk@umb.edu.pl (B.P.); renata.posmyk@umb.edu.pl (R.P.); 2 Podlaskie Center of Clinical Genetics “Genetics”, 15-224 Bialystok, Poland

**Keywords:** hereditary breast cancer, *BRCA1*, *BRCA2*, founder mutations

## Abstract

Breast cancer (BC) remains one of the most prevalent malignancies worldwide, and genetic factors may influence its development. Approximately 10–15% of all BCs are hereditary and known as Hereditary Breast Cancer (HBC). A remarkable family history and young onset are the strongest risk factors of HBC. The rapid development of genetic testing techniques has increased the detection rate of pathogenic and likely pathogenic variants in several genes associated with high, moderate, or low risk of HBC. This allowed us to identify the whole family at risk of HBC. Among hereditary cases, pathogenic variants (PVs) in the *BRCA1* and *BRCA2* genes are particularly notable, especially in certain populations where founder mutations (specific genetic variants originating from a common ancestor) are more prevalent. In this article, an overview of the current state of knowledge on HBC is provided, focusing on the frequency of founder mutations in the *BRCA1* and *BRCA2* genes in HBC in Poland compared to other countries. We will also highlight the role of genetic counseling in the diagnosis and treatment of BC, emphasizing its crucial importance in identifying genetic predispositions, selecting appropriate therapeutic strategies, and supporting patients and their families in making informed medical decisions.

## Simple Summary

Hereditary breast cancer (HBC) accounts for a notable proportion of all breast cancer (BC) cases and is strongly associated with pathogenic variants (PVs) in the *BRCA1* and *BRCA2* genes. In several populations, certain *BRCA* PVs occur more frequently due to founder effects, reflecting their origin from a common ancestor and subsequent spread within specific regions. Understanding these population-specific founder mutations is essential for improving diagnostic accuracy, genetic counseling, and personalized surveillance strategies. In Poland, a distinct spectrum of *BRCA1* and *BRCA2* founder mutations has been identified, influencing national testing protocols and allowing more efficient identification of individuals at high genetic risk. This review summarizes current knowledge on the prevalence of founder mutations in Poland compared with neighboring countries, highlights their clinical implications, and emphasizes the crucial role of genetic counseling in prevention, risk assessment, and therapeutic decision-making for hereditary breast cancer.

## 1. Introduction

Breast cancer (BC) is the second most commonly diagnosed malignancy worldwide, behind only lung cancer. In women, BC is the most common type of cancer on a global level [1]. In 2022, BC affected 2.3 million women worldwide, resulting in 670,000 deaths. In developed countries, one in eight women will develop BC, and the average age of diagnosis is about 61 years [2]. Europe accounts for 24.3% of all BC cases [3]. In Poland, the crude rate of BC is 107.40 per 100,000, which is relatively low by European standards [1,4]. A significant increase in the incidence has been observed in recent decades, which is associated with both changes in women’s lifestyles and advances in diagnostic techniques that allow earlier detection of breast cancer.

**Figure 1:**
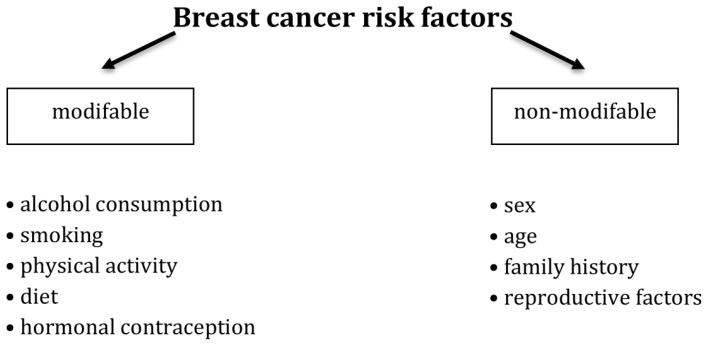
Schematic representation of breast cancer risk factors.

Despite expanding knowledge about BC over the years, scientists are still unable to determine the main cause of breast cancer conclusively. However, much research on BC has isolated risk factors that may increase the incidence of BC. These factors can be both modifiable and non-modifiable. Factors beyond our control include gender, age, race, and family history of risk. Leading non-modifiable risk factors also include a low number of pregnancies, childlessness, first birth at a late age, a short breastfeeding period, early menarche, and late menopause [5,6,7]. Lifestyle also has a substantial impact on the incidence of BC. Among the modifiable risk factors for BC, we can include: alcohol consumption, smoking, physical activity, diet, and use of contraceptives [8,9,10]. A schematic overview of breast cancer risk factors is presented in Figure 1.

Hereditary breast cancer (HBC) accounts for approximately 5–10% of all BC cases, highlighting its significant role as a distinct subgroup of the disease [11]. Pathogenic variants (PVs) in the *BRCA1* and *BRCA2* genes play a pivotal role in the pathogenesis of HBC and are among the most well-studied and thoroughly characterized. Beyond *BRCA1* and *BRCA2*, other, rarer gene PVs, such as *CHEK2*, *PALB2*, *TP53*, *PTEN*, and *ATM*, also contribute to an increased risk of BC. Each of these genes plays a crucial role in essential cellular processes, including DNA repair, cell growth regulation, and tumor suppression. When mutations occur, these processes are disrupted, raising the risk of cancer. Understanding the full range of gene PVs that contribute to BC risk is crucial for developing effective screening and prevention strategies [12,13,14,15,16,17].

Founder mutations in the *BRCA1* and *BRCA2* genes represent a unique genetic phenomenon observed in various populations worldwide. These mutations are so named because they originate from a common ancestor and are subsequently passed down through generations within a specific population or ethnic group. As a result, certain populations may exhibit a higher frequency of specific *BRCA1* or *BRCA2* mutations, known as founder mutations, compared to the general population. Understanding the prevalence and distribution of these founder mutations is crucial for providing accurate genetic counseling, assessing risk, and implementing targeted screening strategies in affected communities [18,19,20,21].

### Family Risk

Familial risk is a very important factor associated with BC. Research shows that having relatives with breast or ovarian cancer (OC) dramatically increases the risk of developing this cancer. This risk increases proportionally with the number of close relatives (first-degree relatives: mother, sister, daughter) diagnosed with BC. Epidemiological studies have shown that having a close relative with BC doubles the risk of developing the disease compared to the general population. In cases where more than one close relative has breast cancer, this risk increases even further [22,23].

**Figure 2:**
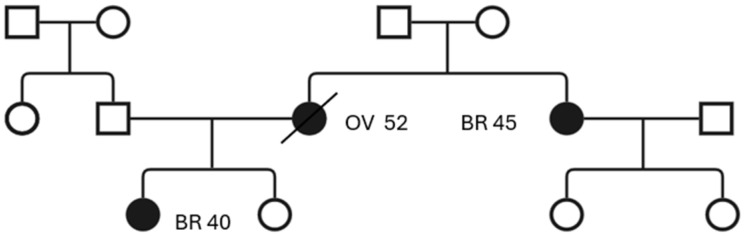
Sample pedigree of a family. The site of cancer and age of diagnosis are indicated next to the symbol. Squares indicate males and circles indicate females; filled symbols represent affected individuals, while open symbols indicate unaffected individuals; symbols crossed with a diagonal line indicate age at diagnosis; numbers indicate age at diagnosis; BR—breast cancer; OV—ovarian cancer.

Data from the Generations Study, which included over 113,000 women from the UK, demonstrated that women with a higher FHS (Family History Score) had a significantly increased risk of developing BC. Specifically, those in the highest FHS group had a 3.5-fold increased risk compared to those with no family history of BC. Additionally, the study found that combining the FHS with the age of the youngest relative diagnosed with BC provided an even stronger predictor of risk [24]. A representative pedigree of a family with breast and ovarian cancer is presented in Figure 2.

## 2. Methodology

Comprehensive electronic literature search was conducted on legitimate databases including PubMed, Web of Science, MEDLINE and Google Scholar using the key-words “hereditary breast cancer”, “breast cancer”, “*BRCA1*”, “*BRCA2*”, “*BRCA* mutation”, “founder mutations”, “*BRCA* mutation in Poland”, and “germline mutations”. Both original research articles and review papers were included. The search covered research papers until 2025 in English. The search identified 281 articles, of which 185 were screened, and 115 were fully read. The findings of the review were synthesized and summarized in a narrative format.

The present review was conducted as a narrative overview of the available literature. While a broad and structured search of multiple databases was performed, the review did not follow a fully systematic methodology with protocol registration (e.g., PROSPERO). Therefore, the findings should be interpreted as a qualitative synthesis of current knowledge, aiming to highlight prevailing trends and population-specific patterns rather than to provide an exhaustive quantitative assessment.

## 3. Hereditary Breast Cancer

**Figure 3:**
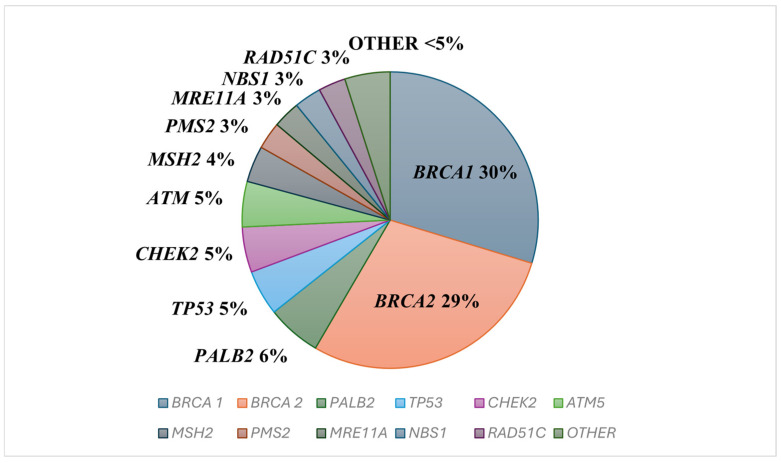
Frequency of pathogenic variants in breast cancer in women [29,30,31,32,33]. Percentages may not sum to 100% due to rounding.

The concept that breast cancer (BC) can be inherited and passed from generation to generation was first described by Paul Broca in 1866 [25]. In 1994 and 1995, respectively, the two major BC susceptibility genes *BRCA1* and *BRCA2* were discovered, confirming the link between family history and the presence of hereditary genetic events that predispose individuals to develop BC [26,27]. In the past two decades, since the discovery of the *BRCA* genes, significant progress has been made in identifying further pathogenic germline variants (PVs) in BC susceptibility genes. A substantial proportion of hereditary breast cancer (HBC) cases are due to germline PVs in the *BRCA1* and *BRCA2* genes, which are responsible for the highest risk of BC. Germline PVs in other genes, such as *TP53*, *NBN*, *PALB2*, *CHEK2*, *PTEN*, *CDH1*, and ATM, are also associated with a high or intermediate risk of BC [28]. The distribution of germline pathogenic variants across breast cancer susceptibility genes is summarized in Figure 3.

### 3.1. Location and Function of BRCA1/2 Genes

The *BRCA1* gene is situated on the long arm of chromosome 17 at *locus* 17q21. It spans approximately 81 kilobases of genomic DNA and includes 24 exons that code for a protein consisting of 1863 amino acids [26,34,35].

The *BRCA2* gene is located on the long arm of chromosome 13 at *locus* 13q12.3. It encompasses approximately 84 kilobases of genomic DNA and comprises 27 exons, encoding a protein of 3418 amino acids [34,36].

*BRCA1* and *BRCA2* are crucial tumor suppressor genes whose PVs are strongly associated with hereditary breast and ovarian cancers (HBOC). Their functions span DNA repair, transcriptional regulation, and cell cycle control, playing an integral role in maintaining genomic stability [35,37,38,39,40].

### 3.2. Cancer Risk in BRCA1/2 Pathogenic Variant Carriers

Pathogenic variants (PVs) in the *BRCA1* and *BRCA2* genes significantly increase the risk of various cancers. The most well-established associations of *BRCA1* and *BRCA2* PVs are with breast and ovarian cancers (OC). Women with *BRCA1* PVs have a 46–87% lifetime risk of BC and a 39–63% risk of OC. Similarly, *BRCA2* PVs confer a 38–84% risk of BC and a 16.5–27% risk of OC. In addition to breast and ovarian cancer, *BRCA1* and *BRCA2* PVs are also associated with an increased risk of other malignancies, including pancreatic, prostate, and stomach cancers [41,42,43,44,45,46,47].

Another study of 3886 women shows that the risk of developing BC by the age of 80 is approximately 72% for *BRCA1* PV carriers and about 69% for *BRCA2* PV carriers [48].

Both *BRCA1* and *BRCA2* PVs are linked to an increased risk of male BC, although the risk is higher for *BRCA2* PV carriers. Men with *BRCA2* PVs have a relative risk (RR) of 44.0, corresponding to a cumulative risk of up to 3.8% by the age of 80, whereas *BRCA1* PV carriers have an RR of 4.30 and a cumulative risk of approximately 0.4% [43].

## 4. Prevalence of *BRCA1* Founder Mutations

The frequency of heterozygous carriers of germline pathogenic variants (PVs) in the *BRCA1* gene in the general White population is estimated to be between 1/500 [49] and 1/1000 [50]. Mutations in the *BRCA1* gene are scattered along the entire coding sequence. Among the identified alterations are deletions and insertions (~79%), which lead to a change in the reading frame; loss-of-function mutations (~20%), resulting in premature translation termination and the formation of a truncated protein; and sense-change mutations (up to 5%). Some of these mutations are common, while others are rare, unique, or are so-called polymorphisms of as yet unproven pathogenicity.

According to the *Breast Cancer Information Core database (BIC)* [51], approximately 2000 different *BRCA1* gene alterations have been recognized to date.

It has been observed that in certain populations with a specific geographical location or ethnicity, certain mutations recur relatively frequently, which is related to the so-called founder effect. Examples of such variations are mutations found in Iceland [52], the Bahamas [53], the French province of Canada (Quebec) [54], Denmark [55], Sweden [56] or Norway [57]. According to *BIC* data, two mutations predominate among all mutations within the *BRCA1* gene, namely c.68_69delAG (185delAG) in exon 2 (59%) and c.5266dupC (5382insC) in exon 20 (31%) [51]. Both of these alterations are considered founder mutations among the most homogeneous group of Ashkenazi Jews (ASH) [58,59]. It has been calculated that in the general population of the Ashkenazi Jews, the c.68_69delAG (185delAG) mutation occurs at a frequency of 1.09% [60], and the c.5266dupC (5382insC) mutation was found in 0.13% [61]. The c.5266dupC (5382insC) mutation is frequently found in Central and Eastern Europe (Poland, Lithuania, Belarus, Ukraine, Russia, Germany), where the ancestors of the almost ten million Ashkenazi Jewish population now living around the world originated [62,63]. Neuhausen et al. (1996), based on a haplotype and phenotype study of six different mutations in the *BRCA1* gene in 61 families, proved that the c.5266dupC (5382insC) mutation originated in the present-day territories of the Baltic countries and spread deep into Europe and the United States with the migration of populations, particularly those with Ashkenazi roots [64].

### 4.1. Poland

Thanks to many years of research conducted by a group of scientists from Szczecin, it is now known that there are three founder mutations in Poland: c.5266dupC (5382insC) in exon 20, c.181T>G (300T/G) in exon 5, and c.4035delA (4153delA) in exon 11. They are responsible for up to 91% of all mutations within the *BRCA1* gene [65]. Thanks to this discovery, the cost of expensive DNA testing has decreased considerably, and screening targeting only three founder mutations is now available to a broader range of breast and/or ovarian cancer (OC) patients, as well as their relatives. In 2014, three new ones were identified by Szwiec M et al., who identified recurrent mutations in *BRCA1*: c.3700_3704del (3819del5), c.68_69delAG (185delAG), and c.5251C>T (5370C>T) were found in multiple families, prompting the recommendation to expand the standard testing panel to include these mutations. This extended panel increased the detection rate of *BRCA* PVs from 5.2% to 6.7% [66].

**Figure 4:**
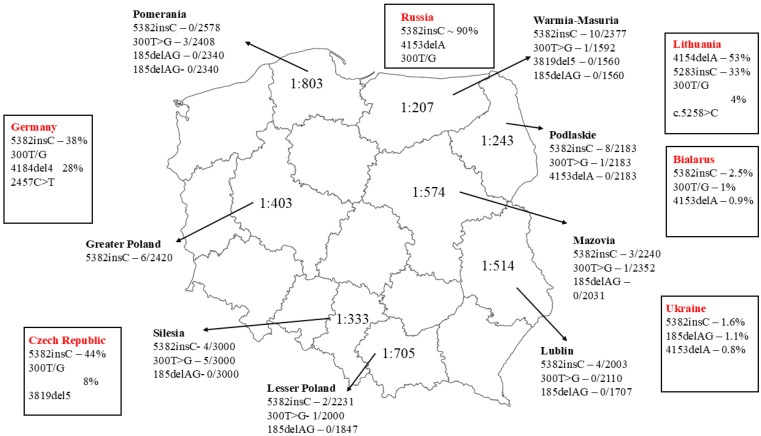
Prevalence of 5382insC, 300T/G, 185delAG and 3819del5 mutations in the *BRCA1* gene in newborns from seven regions of Poland [67] and the most frequent mutations in Poland’s neighbors.

Several large studies on the prevalence of founder mutations in the *BRCA1* gene have been conducted in the general Polish population. The largest number of DNA samples from anonymous newborns from seven regions of Poland (Pomerania, Warmia and Mazury, Mazovia, Greater Poland, Lesser Poland, Lublin and Silesia) was studied by Brożek et al. [67]. The frequency of the c.5266dupC (5382insC) mutation was calculated to be 29/16,849 (0.17%) and that of the c.181T>G (300T/G) mutation to be 11/13,462 (0.08%) [67]. The third founder mutation c.4035delA (4153delA) was not investigated due to the fact that it had not been found in previous reports from these regions [68,69,70,71,72]. The authors noted statistically significant differences in the frequency of individual mutations in newborns from different regions of Poland. The c.5266dupC (5382insC) mutation was most frequently recorded in Warmia and Mazury (10/2377; 0.42%), in contrast to Pomerania (0/2578; 0.0%) (*p* = 0.001) and Lesser Poland (2/2231; 0.09%) (*p* = 0.028). The 3819delA mutation was found to be recurrent, occurring at a frequency of 4/2363 (0.17%), but only in Pomerania. In contrast, it was not found in the neighboring Warmian-Masurian province: 0/1560 [72]. The regional distribution of the most frequent *BRCA1* founder mutations in Poland and neighboring countries is shown in Figure 4.

The 5382insC mutation in exon 20 of the *BRCA1* gene is the most frequently described alteration in Europe (according to *BIC* data). Sobczak et al. [73]. were the first to describe this alteration as frequent among families burdened with breast cancer (*HBC*) and/or ovarian cancer (*HOC*, *HBOC*) in Poland [73]. The frequency of this mutation among families with *HBC*, *HOC* or *HBOC* in Poland is 34% [74], in Russia—14% [75], in Hungary—14% [76], in Slovenia—13% [77], among Ashkenazi Jewish—10% [78], in Germans—4% [79]. In contrast, it has not yet been found among Spaniards, Portuguese, Belgians, Dutch, and Scandinavians [19]. Among Russians, Belarusians, Poles, Latvians, Czechs, and Lithuanians, this mutation accounted for 94% [80], 73% [81], 60% [74], 55% [82], 37–52% [83,84], and 34% [85] of all pathogenic variants (PVs) detected in the *BRCA1* gene.

The c.181T>G (300T/G) mutation in exon 5 was first recognized in 1994 in families of Polish, Russian, and German origin [86]. Since then, it has been reported in many countries, including Austria [87], Germany [79,88], Latvia [89], and other neighboring countries listed below.

Kozlowski et al. [90] and Sobczak et al. [73] were the first to identify the c.4035delA (4153delA) mutation in exon 11 in Poland [73,90]. Subsequently, this mutation was described as frequent in Russia [91], in north-western Poland [65], and in the Baltic countries, where this alteration predominates as a distinct founder effect (Table 1).

### 4.2. Other Countries

#### 4.2.1. Belarus

In the general population of BC patients, the prevalence of *BRCA1* gene PVs was calculated to be 4.4%. In the control group (general population), the frequency of PVs was 0.5%. Three founder mutations were identified for this particular population: c.5266dupC (5382insC) (2.5%), c.181T>G (300T/G) (1%), and c.4035delA (4153delA) (0.9%) [99]. Uglanitsa et al. (2010) [100] identified 38 of the above PVs in the *BRCA1* gene among a group of 500 unselected breast cancers (7.6%). Mutations were more common in women < 50 years of age (12.6%) than in women > 50 years of age (5.6%). In the neonatal control group, the frequency was 2/251 (0.8%) [100].

#### 4.2.2. Ukrainerus

Kitsera et al. [101] studied 335 women with breast cancer from the Western Ukraine region. However, 36 of them (10.7%) had at least a first-degree relative with breast cancer. A total of 125 affected women with an identified family history of BC were eligible for DNA testing. In 5 cases (4%), PVs in the *BRCA1* gene were identified: c.5266dupC (5382insC)—2/125 (1.6%), c.68_69delAG (185delAG)—2/125 (1.6%), and c.4035delA (4153delA)—1/125 (0.8%) [101].

Similar findings were reported by Nguyen-Dumont et al. Among Ukrainian women, the prevalence of founder mutations was 13% (16 out of 123). Eleven carriers of the *BRCA1* 5382insC (c.5266dup) mutation, three carriers of the c.181T>G (181T>G) mutation, and two carriers of the c.68_69del (85delAG) mutation were identified. The c.4035del (4153delA) mutation was not observed in this Ukrainian cohort [92].

#### 4.2.3. Czech Republic

Three founder mutations were found in the Czech population—c.181T>G (300T/G), c.5266dupC (5382insC), c.3700_3704del5 (3819del5)—which accounted for 52% of all detected PVs in the *BRCA1* gene in the population-based study, with the most frequent mutation being c.5266dupC (5382insC) (44%) [84]. A strong Slavic founder effect was observed, especially for two mutations in the *BRCA1* gene: c.181T>G (300T/G) and c.5266dupC (5382insC), which are also considered founder mutations in Poland and other Slavic countries [65].

#### 4.2.4. Estonia

As reported by Tamboom et al. [102], c.5266dupC (5382insC) was the most common mutation in Estonia, with c.4035delA (4153delA) being the second most common [102].

#### 4.2.5. Latvia

In Latvia, the two mutations c.5266dupC (5382insC) and c.4035delA (4153delA) account for more than 80 per cent of all *BRCA1* gene alterations detected. The third—c.181T>G (300T/G)—is recurrent, but at a lower frequency. The c.4035delA (4153delA) mutation is most common among the indigenous Baltic population [82,103].

#### 4.2.6. Lithuania

A study of PV carriage in the *BRCA1* gene in Lithuania shows that there is a strong Baltic founder effect in this country, with the c.4035delA (4153delA) mutation dominating (53%), as in Latvia and Estonia, followed by the c.5266dupC (5382insC) mutation with 33% [104]. Two further mutations c.181T>G (300T/G) and c.5258G>C (5377G>C/R1753T) were also found, which together accounted for 4% of all detected lesions [85,105].

#### 4.2.7. Germany

Eighteen PVs were detected in Germany, including the four most frequent—c.5266dupC (5382insC), c.181T>G (300T/G), c.4065_4068del4 (4184del4), and c.2338C>T (2457C>T)—which together accounted for 66% of all lesions. Mutations c.5266dupC (5382insC) and c.181T>G (300T/G) accounted for 38% [49,106].

#### 4.2.8. Russia

In Russia, the c.5266dupC (5382insC) mutation is the vast majority (~90%). c.4035delA (4153delA), c.181T>G (300T/G) and c.68_69delAG (185delAG) are listed as the next most frequent in Western Russia [75,80,107,108].

#### 4.2.9. Italy

Studies from Southern Italy indicate that the distribution of *BRCA1* pathogenic variants is characterized by marked regional heterogeneity. Importantly, the *BRCA1* c.5266dupC (5382insC) pathogenic variant has been identified in several unrelated hereditary breast and ovarian cancer families from Western Sicily, with a clear geographical clustering in areas surrounding Palermo on the northern coast and Agrigento on the southern coast of the island. This observation suggests that, beyond its well-established presence in Central and Eastern Europe, the c.5266dupC (5382insC) variant also contributes to the hereditary breast and ovarian cancer burden in selected regions of Southern Italy, likely reflecting historical migration and population admixture in the Mediterranean area. In addition to c.5266dupC (5382insC), other *BRCA1* pathogenic variants have been reported in Italian populations, including region-specific founder alterations such as c.4964_4982del (5083del19), as well as less frequent recurrent variants observed sporadically across different regions of Italy, further supporting the presence of population-specific founder effects [109,110].

## 5. Prevalence of *BRCA2* Founder Mutations

The frequency of heterozygous germline pathogenic variants (PVs) in the *BRCA2* gene in the general White population is estimated to range from 1/200 to 1/700 [50,111].

Similar to certain mutations in the *BRCA1* gene, specific mutations in *BRCA2* may exhibit a founder effect. An example is the founder mutation 999del5 in the *BRCA2* gene, which occurs in the Icelandic population with a frequency of approximately 0.4% and accounts for 8.5% of breast cancer (BC) cases [52,112]. In Finland, the mutations 9345+1G>A, c.7480C>T (C7708T), and c.8327T>G (T8555G) are specific to this population, while the mutation 8128delA is considered a founder mutation in the Bahamas [113,114]. Among one of the most genetically homogeneous groups, Ashkenazi Jews, the founder mutation in the *BRCA2* gene is c.5946delT (6174delT), occurring with a frequency of 1.52% in this population [61,115].

### 5.1. Poland

In the Polish population, a wide variety of PVs in the *BRCA2* gene has been observed. Specific recurrent *BRCA2* mutations have been reported; however, their frequency in Polish families is relatively low [93]. Nevertheless, several studies have been conducted in Poland on *BRCA2* gene PVs that increase the predisposition to BC.

According to the study by Cybulski et al., among 144 women with BC, 12 PVs in the *BRCA2* gene were detected (8.3%). Recurrent mutations were identified in individual patients, including c.8647delC (8875delC), c.9402delC, c.9089_9090insA, c.2T>C, c.6275_6276delTT (6503delTT), c.2806_2809delAAAC (3034del4), c.5718_5719delCT, c.9246_9247insA, and c.6402_6406delTAACT (6630del5) [94].

A study conducted on a group of 1164 women with BC, recruited from two centers—Szczecin and Opole—revealed the presence of five distinct mutations in the *BRCA2* gene. In the Opole center, mutations c.658_659delGT (886delGT) and c.3847_3848delGT (4075delGT) were identified, while in the Szczecin center, mutations c.5239_5240insT (5467insT), c.5946delT (6174delT), and c.7913_7917del5 (8138del5) were detected. Each of these mutations was observed in a single case [66]. The mutations detected in Szczecin were also identified as recurrent in studies conducted by Gaj et al., with frequencies of 4, 4, and 2, respectively. Overall, *BRCA2* PVs were detected in 12 out of 906 individuals, representing 1.3% of the study population [95]. In a study by Balabas et al., both the c.5239_5240insT (5467insT) and c.5946delT (6174delT) mutations were each detected once among 105 individuals, 91 of whom had BC; the c.5239_5240insT (5467insT) variant was identified in a male patient [116]. The c.5946delT (6174delT) variant is a frequently occurring mutation in the Ashkenazi Jewish population [117]. Additionally, the c.9098_9099insA (9326insA) mutation, also identified in the studied population, was previously reported in the German population [106,116].

In northeastern Poland, recurrent mutations such as c.3847del (4075delG) and c.3860delA (4088delA) (the latter likely originating from Spain or Western Europe) were observed, along with newly identified mutations 7327ins/dupl19, 9068del, and 7985G>A [68,71].

A study conducted in southeastern Poland among 121 women with BC and/or OC, of whom 115 had BC, identified seven *BRCA2* PVs that were present only in women with BC. The mutations c.1310_1313delAAGA (1529delA), c.9371A>T, and c.9402delC (9631delC) were each identified twice [93]. Other studies conducted in the same region of Poland also identified the c.9402del (9631del) mutation in three unrelated families among the 47 families studied with BC and/or OC from Upper Silesia, along with one occurrence of the 6886del5 mutation [118]. The c.9402del (9631del) mutation has also been reported in patients from the Czech Republic and Slovakia, suggesting it may represent a local founder variant [119,120].

In a group of 512 patients with BC and/or OC from the Warsaw Oncology Center, whose blood samples were collected between 2003 and 2010, Kluska et al. identified a total of 52 (10%) PVs in the *BRCA1/2* genes. The mutations c.7251_7252delCA (7477delCA) and c.9118-2A>G (IVS23-2A>G) were each detected in three patients, while the c.9371A>T (N3124I) mutation, classified as a variant of uncertain significance (VUS), was identified in six patients. The remaining pathogenic mutations were found as single occurrences [96].

Studies conducted on a group of 2466 women with early-onset BC revealed that *BRCA2* PVs were rare in this population, occurring in only 0.08% of patients diagnosed with BC at age 40 or younger. Only two individual *BRCA2* mutations were identified: c.3847_3848delGT (4065delGT) and c.658_659delGT (680delGT) [97] (Table 2).

### 5.2. Other Countries

#### 5.2.1. Belarus

Reports on *BRCA2* mutations in Belarus are limited, which means that our understanding of the full spectrum of these mutations remains incomplete. In a study involving 340 patients with hereditary breast and ovarian cancer (HBOC), pathogenic variants in the *BRCA1/2* genes were found in 98 patients (29%). Among the Belarusian population, the founder of the *BRCA2* c.658_659delGT (886delGT) mutation was identified in three cases [124].

#### 5.2.2. Russia

Data on *BRCA2* mutations in Russia is limited. In Russia, the vast majority of *BRCA2* mutations consist of the Ashkenazi variant c.5964delT (6174delT), which may be due to the long-standing presence of the Jewish community in Russia and its high level of integration with the native population [108].

#### 5.2.3. Lithuania

In the Lithuanian population, *BRCA2* mutations are less common compared to *BRCA1* mutations, but their presence is significant in the context of HBOC risk. The most frequently occurring founder mutation in the *BRCA2* gene in this population is c.658_659del (886delGT), which accounts for half of all *BRCA2* mutations and 5% of all *BRCA1* and *BRCA2* mutations combined. This mutation is likely associated with a founder effect, as it has not been widely reported in other populations, suggesting its specificity to this region [105].

#### 5.2.4. Czech Republic

In studies conducted among Czech patients at high risk or suffering from BC and/or OC, a founder effect was observed for two *BRCA2* mutations—c.7913_7917del5 (8138del5) and c.8537_8538del2—which have only been reported in Canada and the USA [84,125]. These mutations accounted for 15.6% and 16.7% of all identified *BRCA2* mutations, respectively. Additionally, Foretova et al. demonstrated in their research that another common *BRCA2* mutation, c.7910_7914del5, represented 26% of the identified *BRCA2* PVs [119].

#### 5.2.5. Germany

In Germany, 13 recurrent *BRCA2* PVs were identified, accounting for 44% of all mutations in this gene. Seven of these mutations, representing 28% of all identified mutations, occurred at least three times. The most common mutations showing a likely founder effect include: c.1813dupA (2041insA), c.4478del4 (4706del4), and c.9098dupA (9326insA) [106].

#### 5.2.6. Ukraine, Estonia, Latvia

Currently, there is no compelling data on *BRCA2* founder mutations in these populations.

## 6. Genetic Counseling

Genetic counseling for breast cancer (BC) has become increasingly important with advances in genetic testing and understanding of hereditary cancer syndromes. This counseling helps identify individuals at high risk for BC based on the family history and genetic composition, providing them with personalized risk management strategies and informed decision-making tools [126].

### 6.1. Impact of Genetic Counseling

Genetic counseling and testing enable the development of tailored risk management strategies. High-risk individuals may benefit from increased surveillance, including more frequent mammograms and MRI scans, and preventive options such as prophylactic mastectomy and oophorectomy. For those already diagnosed with BC, genetic testing can inform treatment decisions. For instance, *BRCA1/2* pathogenic variant (PV) carriers may be eligible for PARP inhibitors, which have shown efficacy in treating certain types of BC. Genetic counseling also extends to family members. Identifying a pathogenic variant in an individual can prompt testing and preventive measures for relatives, potentially reducing the overall familial cancer burden [47,126].

### 6.2. Challenges and Future Directions

Despite the benefits, there are challenges in genetic counseling for BC. These include disparities in access to genetic services, particularly in underserved populations, as well as the need for a greater number of trained genetic counselors. Furthermore, the interpretation of variants of unknown significance (VUS) remains complex and can lead to uncertainties in clinical management. Advancements in technology, such as next-generation sequencing, have made multigene panel testing more accessible and comprehensive. However, this also increases the likelihood of detecting VUS, necessitating ongoing research and updated guidelines to manage these findings effectively.

In the future, integrating genetic counseling into routine oncology practice and conducting longitudinal studies to understand the long-term outcomes of genetic testing will be crucial. These efforts will help ensure that all individuals at risk for hereditary BC receive the benefits of genetic counseling and testing [21].

## 7. Conclusions

The findings presented in this study highlight the significant role of founder mutations in the *BRCA1* and *BRCA2* genes in hereditary breast cancer (HBC), particularly within the Polish population. The identification of specific recurrent mutations, such as 5382insC, 300T/G, and 4153delA, underscores the importance of tailored genetic testing strategies to improve diagnostic efficiency and accessibility. Comparative analysis with other populations demonstrates the impact of geographical and ethnic factors on PV prevalence, emphasizing the need for population-specific screening protocols.

Additionally, the integration of genetic counseling into cancer management plays a critical role in risk assessment, treatment planning, and preventive measures for individuals and families affected by hereditary BC. Future research should focus on expanding the understanding of genetic variants, addressing challenges in the interpretation of variants of uncertain significance, and promoting equitable access to genetic services. These efforts will enhance the early detection and management of hereditary BC, ultimately reducing its burden on affected populations.

## Figures and Tables

**Table 1:** Mutations in the *BRCA1* gene identified in Polish population [65,66,67,71,72,73,74,92,93,94,95,96,97,98]. N—number of families; n—number of probands.

	Sobczak [73]	Górski [65]	Perkowska [71]	Górski [74]	Lubiński [98]	Ratajska [72]	Brożek [67]	Gaj [95]	Cybulski [94]	Szwiec [66]	Kluska [96]	Wójcik [93]	Rogoza-Janiszewska [97]	Nguyen-Dumont [92]
	n = 236	N = 66	N = 60	N = 200	n = 3472	N = 64	n = 1185	n = 906	n = 144	n = 1164	n = 512	n = 121	n = 2464	n = 337
c.5266dupC (Polish founder mutation)	-	18	6	68	134	17	35	114	-	39	-	6	184	24
c.181T>G (Polish founder mutation)	-	7	3	31	52	9	18	38	-	19	-	4	83	7
c.4035delA (Polish founder mutation)	1	4	-	12	12	-	-	2	-	3	-	-	13	2
c.68_69delAG	-	2	1	1	-	3	-	8	-	5	-	-	6	1
c.5251C>T	-	-	-	1	-	-	-	8	1	3	-	-	5	1
c.4501C>T	-	-	-	-	-	-	-	-	-	-	1	-	-	-
c.190T>C	-	1	-	-	-	-	-	-	-	-	-	-	-	-
c.2563C>T	-	-	1	-	-	-	-	-	-	-	-	--	-	-
c.2872_2876delTTTCA	-	-	-	-	-	1	-	-	-	-	-	-	-	-
c.4357+1G>A	-	-	1	-	-	-	-	-	-	-	-	-	-	-
c.4516delG	-	-	-	-	-	-	-	-	-	-	4	-	-	-
c.2866_2870delTCTCA	-	-	-	1	-	-	-	-	-	-	-	-	-	-
c.4243G>T	-	-	-	-	-	-	-	-	-	-	1	-	-	-
c.403delAAGA	-	-	-	-	-	-	-	-	-	-	1	-	-	-
c.3700_3704del5	-	2	1	3	-	4	-	11	-	9	-	-	9	-
c.3817C>T	-	-	-	-	-	1	-	-	-	-	-	-	-	-
c.340delTC	-	-	-	-	-	-	-	-	-	-	1	-	-	-
c.675delT	-	-	-	2	-	-	-	-	-	-	-	-	-	-
c.3756_3759delGTCT	-	-	-	-	-	-	-	4	-	-	-	--	-	-
c.5285insG	-	-	-	-	-	-	-	-	-	-	1	-	-	-
c.5344G>A	-	-	1	-	-	-	-	-	-	-	-	-	-	-
c.3779delT	-	-	-	-	-	-	-	2	-	-	-	-	-	-
c.403delA	-	-	-	-	-	-	-	-	-	-	1	-	-	-
c.5028_5031delGAAA	-	-	-	1	-	-	-	-	-	-	-	-	-	-
c.4041_4042delAG	-	-	-	-	-	-	-	1	1	-	-	-	-	-
c.5211G>A	-	-	-	-	-	-	-	1	-	-	-	-	-	-
c.1612_1616del	-	-	-	-	-	-	-	-	-	-	-	-	-	1
c.1695insG	-	-	-	-	-	-	-	-	-	-	1	-	-	-
c.5177_5180delGAAA	-	-	-	-	-	-	-	-	1	-	-	-	-	-
c.5346G>A	1	-	-	-	-	-	-	-	1	-	4	-	1	1
c.1687C>T	-	-	-	-	-	-	-	-	1	-	8	-	-	-
c.4689C>G	-	-	-	-	-	-	-	-	-	-	1	-	-	-
c.843_846del	-	-	-	-	-	-	-	-	-	-	-	-	-	1
c.4597delGA	-	-	-	-	-	-	-	-	-	-	1	-	-	-
c.5232del7ins12	-	-	-	-	-	-	-	-	-	-	1	-	-	-
c.1612C>T	-	-	-	-	-	-	-	-	-	-	1	-	-	-
c.5137delG	-	-	-	-	-	-	-	-	-	-	1	-	-	-
c.3531delT	-	-	-	-	-	-	-	-	-	-	-	1	-	-
c.314A>G	1	-	-	-	-	-	-	-	-	-	-	-	-	-
c.374dup	-	-	-	-	-	-	-	-	-	-	-	-	-	1

**Table 2:** Mutations in the *BRCA2* gene identified in Polish population [66,68,71,72,74,92,93,94,95,96,97,116,118,121,122,123].

	Looij [68]	Grzybowska [118]	Kwiatkowska [121]	Perkowska [71]	Górski [74]	Brożek [122]	Ratajska [72]	Balabas [116]	Gaj [95]	Cybulski [94]	Ratajska [123]	Szwiec [66]	Kluska [96]	Wójcik [93]	Rogoża-Jerzewska [97]	Nguyen-Dumont [92]	Total
	n = 25	n = 47	n = 37	n = 60	n = 200	n = 151	n = 64	n = 105	n = 906	n = 144	n = 134	n = 1164	n = 512	n = 121	n = 2464	n = 337	
c.2 T>C	-	-	-	-	-	-	-	-	-	1	-	-	-	-	-	-	1
c.22_23delAG	-	-	-	-	-	-	-	-	-	1	-	-	-	-	-	-	1
c.262_263delCT	-	-	-	-	1	-	-	-	-	-	-	-	-	-	-	-	1
c.274C>T	-	-	-	-	-	-	-	-	-	-	-	-	1	-	-	-	1
c.658_659delGT	-	-	-	-	-	-	-	-	-	-	-	1	-	-	1	-	2
c.700delT	-	-	-	-	-	-	-	-	-	-	-	-	1	-	-	-	1
c.1180G>T	-	-	-	-	-	-	-	1	1	-	-	-	-	-	-	-	2
c.1310_1313delAAGA	-	-	-	-	-	-	-	-	-	-	-	-	-	2	-	-	2
c.1318_1321delCTTA	-	-	-	-	-	-	-	-	-	-	-	-	1	-	-	-	1
c.1813_1814insA	-	-	1	-	-	-	-	-	-	-	-	-	-	-	-	-	1
c.2806_2809delAAAC	-	-	-	-	-	-	-	-	-	1	-	-	-	-	-	-	1
c.2808_2811delACAA	-	-	-	-	-	-	-	-	-	-	1	-	1	-	-	-	2
c.3075_3076delinsTT	-	-	-	-	-	-	-	-	-	-	-	-	-	-	-	1	1
c.3199delA	-	-	-	-	1	-	-	-	-	-	-	-	-	-	-	-	1
c.3847_3848delGT	1	-	-	-	-	-	-	-	-	-	-	1	-	-	1	-	3
c.3860delA	-	-	-	1	-	-	-	-	-	-	-	-	-	-	-	-	1
c.3975_3981dupTGCT	-	-	-	-	-	1	-	-	-	1	1	-	-	-	-	-	3
c.4223delA	-	-	-	-	-	1	-	-	-	-	-	-	-	-	-	-	1
c.5042_5043delTG	-	-	-	-	-	-	-	-	-	-	1	-	-	-	-		1
c.5239_5240insT	-	-	-	-	1	-	-	1	4	-	-	1	-	-	-	1	8
c.5718_5719delCT	-	-	-	-	-	-	-	-	-	1	-	-	-	-	-	-	1
c.5946delT	-	-	-	-	-	-	-	1	4	-	-	1	-	-	-	-	6
c.5964_5965delAT	-	-	-	-	-	-	-	1	-	-	-	-	-	-	-	-	1
c.6010_6030delinsTT	-	-	-	-	-	-	1	-	-	-	-	-	-	-	-	-	1
c.6267_6269delGCAinsC	-	-	1	-	-	-	-	-	-	-	-	-	-	1	-	-	2
c.6275_6276delTT	-	-	-	-	-	-	-	-	-	1	-	-	-	-	-	-	1
c.6393_6396delATTA	-	-	-	-	-	1	-	-	-	-	-	-	-	-	-	-	1
c.6402_6406delTAACT	-	-	-	-	-	-	-	-	-	1	-	-	-	-	-	-	1
c.6447_6448delTA	-	-	-	-	-	-	-	1	-	-	-	-	-	-	-	-	1
c.6658_6662delGAAAA	-	1	-	-	-	-	-	-	-	-	-	-	-	-	-	-	1
c.7007G>A	-	-	-	-	1	-	-	-	-	-	-	-	-	-	-	-	1
c.7180A>T	-	-	-	-	-	-	-	-	-	-	1	-	-	-	-	-	1
c.7251_7252delCA	-	-	-	-	-	-	-	-	-	1	-	-	3	-	-	-	4
c.7080_7099dup	1	-	-	-	-	-	-	-	-	-	-	-	-	-	-	1	2
c.7913_7917delTTCCT	-	-	1	-	1	-	-	3	2	-	-	1	-	1	-	-	9
c.8648delC	-	-	-	-	-	-	1	-	-	1	-	-	-	-	-	-	2
c.8842delA	1	-	-	-	1	-	-	-	-	-	-	-	1	-	-	-	3
c.8924delT	-	-	-	-	-	-	-	1	1	-	-	-	-	-	-	-	2
c.8946delA	-	-	-	-	-	-	-	-	-	-	-	-	1	-	-	-	1
c.9027delT	-	-	-	-	-	-	-	-	-	-	-	-	-	1	-	-	1
c.9	-	-	-	-	-	-	-	1	-	1	-	-	-	-	-	-	2
c.9118-2A>G	-	-	-	-	-	-	-	1	-	-	-	-	3	-	-	-	4
c.9138C>T	-	-	-	-	-	-	-	-	-	-	-	-	1	-	-	-	1
c.9253_9254insA	-	-	-	-	-	-	-	-	-	1	-	-	-	-	-	1	2
c.9371A>T	-	1	1	-	-	-	-	3	-	-	-	-	6	2	-	-	13
c.9376delC	-	-	-	-	-	-	-	-	-	-	-	-	1	-	-	-	1
c.9402delC	-	3	-	-	1	-	-	1	-	1	-	-	-	2	-	-	8
c.10095delCinsGAATTATATCT	-	-	-	-	-	-	2	1	-	-	-	-	-	1	-	-	4
c.5857G>T	-	-	-	-	-	-	-	-	-	-	-	-	-	-	-	1	1
c.8623G>T	-	-	-	-	-	-	-	-	-	-	-	-	-	-	-	1	1
c.6315_6318del	-	-	-	-	-	-	-	-	-	-	-	-	-	-	-	1	1
c.9089_9090insA	-	-	-	-	-	-	-	-	-	1	-	-	-	-	-	-	1
c.9246_9247insA	-	-	-	-	-	-	-	-	-	1	-	-	-	-	-	-	1

## Data Availability

No new data were created or analyzed in this study.

## Abbreviations

The following abbreviations are used in this manuscript:

BC	Breast cancer
OC	Ovarian cancer
HBC	Hereditary breast cancer
FHS	Family History Score
PVs	Pathogenic variants
RR	Relative risk
BIC	Breast Cancer Information Core
ASH	Ashkenazi Jews
HOC	Hereditary ovarian cancer
HBOC	Hereditary breast and ovarian cancer
VUS	Variant of uncertain significance
